# Perioperative transcutaneous electrical acupoint stimulation (pTEAS) in pain management in major spinal surgery patients

**DOI:** 10.1186/s12871-022-01875-3

**Published:** 2022-11-08

**Authors:** Xinyuan Wu, Jieling Huang, Yuling Zhang, Luying Chen, Yandong Ji, Wuhua Ma, Yuhui Li

**Affiliations:** 1 Department of Anaesthesia, The First Affiliated Hospital of Guangzhou University of Chinese medicine, 510405 Guangzhou, China; 2 The First Clinical Medical School of Guangzhou University of Chinese Medicine, Guangzhou University of Chinese Medicine, 510405 Guangzhou, China

**Keywords:** Perioperative transcutaneous electrical acupoint stimulation (pTEAS), Pain management, Major spinal surgery, Clinical outcome

## Abstract

**Background:**

Lumbar disc herniation is seen in 5–15% of patients with lumbar back pain and is the most common spine disorder demanding surgical correction. Spinal surgery is one of the most effective management for these patients. However, current surgical techniques still present complications such as chronic pain in 10–40% of all patients who underwent lumbar surgery, which has a significant impact on patients’ quality of life. Research studies have shown that transcutaneous electrical acupoint stimulation (TEAS) may reduce the cumulative dosage of intraoperative anesthetics as well as postoperative pain medications in these patients.

**Objective:**

To investigate the effect of pTEAS on pain management and clinical outcome in major spinal surgery patients.

**Methods:**

We conducted a prospective, randomized, double-blind study to verify the effect of pTEAS in improving pain management and clinical outcome after major spinal surgery. Patients (n = 90) who underwent posterior lumbar fusion surgery were randomized into two groups: pTEAS, (n = 45) and Control (n = 45). The pTEAS group received stimulation on acupoints Zusanli (ST.36), Sanyinjiao (SP.6), Taichong (LR.3), and Neiguan (PC.6). The Control group received the same electrode placement but with no electrical output. Postoperative pain scores, intraoperative outcome, perioperative hemodynamics, postoperative nausea and vomiting (PONV), and dizziness were recorded.

**Results:**

Intraoperative outcomes of pTEAS group compared with Control: consumption of remifentanil was significantly lower (*P* < 0.05); heart rate was significantly lower at the end of the operation and after tracheal extubation (*P* < 0.05); and there was lesser blood loss (*P* < 0.05). Postoperative outcomes: lower pain visual analogue scale (VAS) score during the first two days after surgery (*P* < 0.05); and a significantly lower rate of PONV (on postoperative Day-5) and dizziness (on postoperative Day-1 and Day-5) (*P* < 0.05).

**Conclusion:**

pTEAS could manage pain effectively and improve clinical outcomes. It could be used as a complementary technique for short-term pain management, especially in patients undergoing major surgeries.

**Trial registration:**

ChiCTR1800014634, retrospectively registered on 25/01/2018. http://medresman.org/uc/projectsh/projectedit.aspx?proj=183

**Supplementary Information:**

The online version contains supplementary material available at 10.1186/s12871-022-01875-3

## Background

Studies have reported that 75–85% of the global population suffers from lumbar back pain at some point in life [1]. Lumbar back pain occurring during old age is often accompanied by psychosocial distress, physical limitation, sleep disturbances, and depression [2]. Among other causes, lumbar disc herniation is seen in 5–15% of the patients with lumbar back pain [3, 4]. This particular etiology usually results in disability, demands surgical correction, and burdens families and society [5, 6].

Nowadays, more clinicians and patients have turned to spinal surgery as a treatment option, as evidenced by a two-fold increase in spinal surgeries performed in the last fifteen years [7]. However, postoperative complications continue to develop in 10–40% of all patients, including chronic persistent postoperative pain [8], anesthetic complications such as post-operative nausea and vomiting (PONV), positioning complications, acute spinal cord injury, vascular injury, cardiovascular events, pulmonary complications. Persistent pain occurs in over 20% of all post-lumbar surgery patients, creating a healthcare burden in the long run. Previous studies have reported that 0.014–0.2% of patients experienced stroke post-spinal surgery [9, 10], around 13% developed pulmonary complications [11] and on average, lost 0.5-3.0 L of blood during operation [12, 13]. Major intraoperative hemorrhage has been identified as an independent predictive marker to develop postoperative complications such as stroke, myocardial infarction [10], and coagulopathy [14]. Without treatment, complications following spinal surgery may put the patient in severe or even permanent morbidity [15–18]. The existing strategy emphasizes multimodal pharmacologic as well as non-pharmacologic interventions that include using different classes of painkillers to manage pain; avoiding nitrous oxide, neostigmine, and inhalational anesthetics to prevent PONV [19]; and using tranexamic acid, magnesium sulfate or inducing hypotension to reduce hemorrhage [7].

Transcutaneous electrical acupoint stimulation (TEAS) has been shown to relieve postoperative pain, reduce the cumulative dosage of intraoperative anesthetics and minimize general anesthesia-related side effects [20]. According to a meta-analysis of 14 randomized controlled trials (RCTs), TEAS can effectively prevent PONV and dizziness [21]. However, how perioperative transcutaneous electrical acupoint stimulation (pTEAS) in spinal surgery affects the clinical outcome remains unclear. This prospective, randomized, double-blind study was conducted to verify whether pTEAS could improve pain management and clinical outcome after spinal surgery.

### Methods

#### Study

This study was a prospective, randomized, double-blind RCT approved by the local Clinical Research Ethics Committee and was registered at clinicaltrials.gov (chiCTR1800014634, 25/01/2018). Written informed consent was obtained from each patient before enrollment in the study. Three primary outcomes were examined: (1) the VAS score for short-term pain management; (2) the consumption of remifentanil; and (3) the incidence of PONV and dizziness. The secondary outcomes were perioperative hemodynamics, intraoperative blood loss, and postoperative consumption of antiemetics.

### Patients

From March 2016 to February 2017, 90 patients undergoing elective major posterior lumbar spinal surgery for spinal stenosis as well as degenerating intervertebral disc under general anesthesia were assigned into two groups: patients to receive total intravenous anesthesia (Control group, n = 45), and others to receive pTEAS in addition to total intravenous anesthesia (pTEAS group, n = 45).

**Inclusion criteria** are as follows: (1) ASA I – II; (2) between 40 and 70 years of age; (3) diagnosed with lumbar spinal stenosis or lumbar intervertebral disc herniation based on imaging results; and (4) undergoing the operative procedures of posterior lumbar decompression, bone graft fusion, and internal fixation.

**Exclusion criteria** include having a past medical history of malignant tumors or severe cardiovascular disease.

**Elimination criteria** are as follows: (1) uncooperative patients who refuse treatment; (2) intraoperative blood loss of more than half of the total blood volume; (3) serious surgical complications such as the patient falling into a coma.

### Randomization and blinding

The sample size was determined by previous literature. All patients (n = 90) were randomized using a computer-generated number. Numbers 1 to 45 were assigned to the pTEAS group, while numbers 46–90 were assigned to the Control group. All researchers involved in this study were blinded to these groupings. Only the stimulator was aware of the groupings; however, the stimulator was not involved in the process of data collection, processing, and the findings of this project.

### pTEAS

**Fig. 1:**
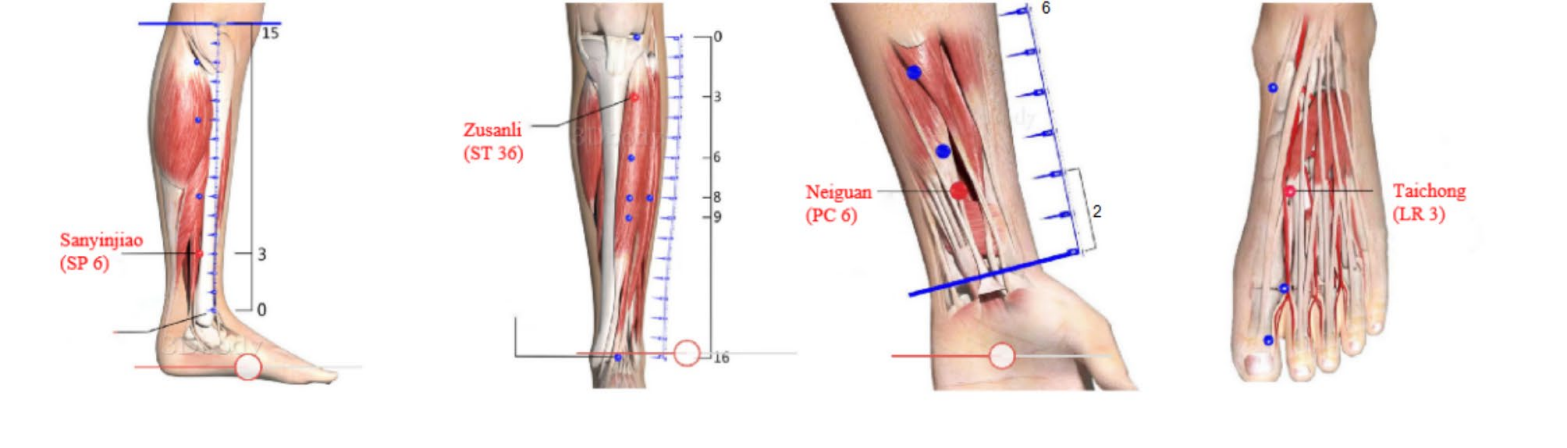
Location of acupoints. From left to right: Sanyinjiao (SP 6). Zusanli (ST 36). Neiguan (PC 6). Taichong (LR 3)

pTEAS was performed on the patient before induction of general anesthesia by a trained doctor. Based on the principle of traditional Chinese medicine, bilateral Zusanli (ST.36) and Sanyinjiao (SP.6) were stimulated by cutaneous electrode pads at 2/15Hz for 30 min before anesthesia induction and then at 2/100Hz throughout the operation. Additional stimulation was administered until the postoperative day (POD) 4, wherein bilateral Taichong (LR.3) and Neiguan (PC.6) were stimulated at 2/15Hz for 30 min once daily. Whereas the stimulator used in the study is Han’s acupoint stimulator (HANS LH-202 H, Huawei Co. Beijing, China), stimulated in a symmetrical bidirectional pulse with a width of 0.2 ~ 0.6 ms, the frequency of the output stimulation is an alternating dense-and disperse-mode of 2/15 or 2/100 Hz, where the 2 Hz (0.6-ms pulse width) stimulation is alternated with 100 Hz stimulation (0.2-ms pulse width) automatically. In each set (2/15 and 2/100), a 3s alternating time was given between 2 and 15 or 2 and 100 Hz, respectively. The intensity was adjusted according to individuals’ maximum tolerance, the instrument we used has two output channels, channel A and channel B. Before anesthesia induction, the anode of channel A is connected to the left Zusanli (ST.36) point, the cathode is connected to the ipsilateral Sanyinjiao (SP.6) point, the anode of channel B is connected to the right Zusanli (ST.36) point, and the cathode is connected to the ipsilateral Sanyinjiao (SP.6) point. During postoperative stimulation, the anode of channel A is connected to the left Taichong (LR.3) point, the cathode is connected to the left Neiguan (PC.6) point, the anode of channel B is connected to the right Taichong (LR.3) point and the cathode is connected to the right Neiguan (PC.6) point. Acupoints were identified in accordance with the traditional anatomic localization (Fig. 1).

The electrodes that we used were silver/silver chloride electrodes produced by Shanghai Shenfeng Medical Care Products Co., Ltd., Shanghai, China, which conduct electricity through the metal material and conductive gel in the middle of the circle and conduct electricity in a circular area with a diameter of 15 mm, and the area of electric conductivity is the “effective area”. The electrodes of each patient are connected to the same acupuncture points in the same way to ensure the same polarity of the electrodes. The acupoint of the pTEAS device was given on patients’ legs or hands that were fully covered by the materials to ensure the pTEAS device was out of sight of blinded researchers. The Control group had the same electrode placement but no electrical stimulation was applied.

### Anesthesia and perioperative management

Under the Target-Controlled Infusion (TCI) system, anesthesia was induced intravenously with propofol and fentanyl. Vecuronium was administered intravenously at 0.1 mg/kg after the patient lost consciousness. Subsequently, an orotracheal intubation tube was placed. Anesthesia was maintained by propofol and remifentanil under the TCI system while the depth of anesthesia was monitored using NarcoTrend Index (NTI). Using the models put forward by Marsh and colleagues [22], the concentrations of propofol (2.5–3.5 µg/ml) and remifentanil (2.0–4.0 ng/ml) in plasma were adjusted according to hemodynamics and NTI (37–64). The TCI pump recorded the cumulative dosage of anesthetics used throughout the operation. The patients received mechanical ventilation set at volume-controlled mode (tidal volume of 6–8 ml/kg according to the intraoperative end-expiratory carbon dioxide controlled between 35 and 45 mmHg). The target-controlled infusion was stopped 10 min before the end of surgery. In both groups, patients were administered with 2 mg/kg tramadol injection and 1 µg/kg fentanyl at the end of the operation before being transferred to the Post-anesthesia Care Unit (PACU) for extubation and recovery.

### Data collection

Perioperative hemodynamics including systolic blood pressure (SBP), diastolic blood pressure (DBP), and heart rate were recorded at five time points: induction, intubation, the start of the operation, end of the operation, and extubation. We calculated the consumption of propofol and remifentanil and recorded the intraoperative blood loss, time to extubation, operation duration, and total anesthesia duration. The patients were followed up until postoperative day (POD) 5 for the visual analogue scale (VAS) score, calculated as follows: 0 represents no pain, 1–3 denotes mild pain, 4–6 signifies moderate pain, > 7 represents severe pain and 10 shows the worst pain possible, as well as the incidence of PONV and dizziness, consumption of rescue antiemetics.

### Statistical analysis

After performing all statistical analyses in SPSS version 19.0, we used the two-sample *t*-test to compare the mean of continuous measurements between study groups. We elucidated the dichotomous variables by ‘the number of patients (percentage)’ and analyzed the data using the chi-square test, Mann–Whitney U test, or Fisher’s exact test. For all statistical tests, the level of significance was set at 0.05.

## Results

**Fig. 2:**
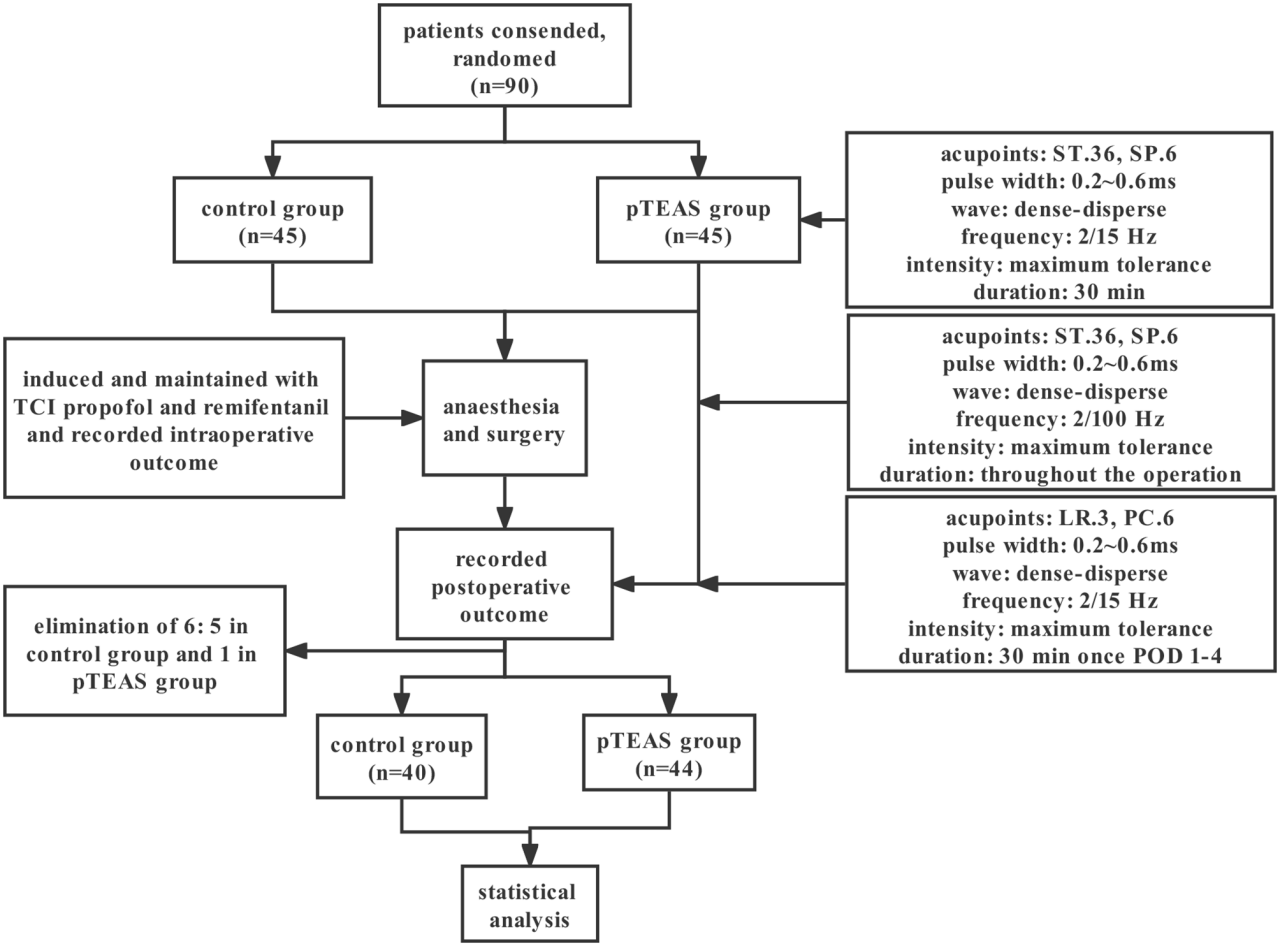
Flow chart depicting the workflow of this trial

Among a total of 90 patients, six were excluded: two patients suffered a massive hemorrhage of more than half of their total blood volume (each group respectively), one patient had a serious postoperative complication (Control group), whereas three patients did not have their data collected completely (Control group). Eventually, 84 patients completed the study; 40 patients in Control group (40/90, 44.4%) and 44 patients in pTEAS group (44/90, 48.9%) (Fig. 2).

The baseline characteristics of both groups were similar to each other (*P* > 0.05) (Table 1).

**Table 1:** Patient characteristic. Data presented as mean (S.D.).

	Control group (n = 40)	pTEAS group (n = 44)
Age (yr)	56.02(8.48)	57.58(7.81)
Sex		
Male	16	10
Female	24	34
Height (cm)	161.53(9.58)	159.11(8.74)
Body Mass Index (BMI)	23.55(2.49)	24.25(3.27)

### Clinical outcome

#### Primary outcome

##### (i) The consumption of remifentanil

Remifentanil, a selective µ-receptor agonist that suppresses somatic stress and adrenergic response, is commonly used in general anesthesia and pain management. However, remifentanil may be associated with opioid-related side effects (including nausea, vomiting, or reduced level of consciousness), causing a delay in postoperative recovery, poor clinical outcome, or other complications such as infection or cancer. According to data gathered from the TCI system, the cumulative dosage and index of remifentanil consumption in the pTEAS group were significantly lower than that in the Control group [Cumulative dosage: 1383(494) vs. 1637(630) µg; Index: 0.084(0.018) vs. 0.114(0.090) µg/min/kg; *P* < 0.05, respectively]. Both groups were similar with respect to cumulative consumption of propofol [(0.086(0.012) vs. 0.091(0.015) mg/min/kg; *P* > 0.05], time to extubation [40.20(19.27) vs. 36.95(16.70) min; *P* > 0.05], duration of surgery [232(70) vs. 215(61) min; *P* > 0.05], and total anesthesia duration [290(71) vs. 271(70) min; *P* > 0.05] (Table 2).

**Table 2:** Consumption of anaesthetics, peri-operative outcomes. Data presented as mean (S.D.).

	Control group (n = 40)	pTEAS group (n = 44)	P-value
Consumption of remifentanil(µg)	1637(630)	1383(494)	0.042^a^
Consumption of propofol(mg)	1529(476)	1478(404)	0.599
Index of propofol Consumption(mg/min*kg^− 1^)	0.086(0.012)	0.091(0.015)	0.064
Index of remifentanil Consumption(µg/min*kg^− 1^)	0.114(0.090)	0.084(0.018)	0.045^a^
Blood loss (ml)	497(283)	379(202)	0.030^a^
Time to extubation (min)	40.20(19.27)	36.95(16.70)	0.411
Operation duration(min)	232(70)	215(61)	0.227
Anaesthesia duration (min)	290(71)	271(70)	0.218

a:P < 0.05 vs. control group

#### (ii) VAS score in pain management

VAS is a measure of the severity of pain based on an individual’s psychometric response. VAS score provides a quick way of categorizing disease severity and determining the appropriate management. We observed the patients until the postoperative day (POD) 5. We found that the VAS score in pTEAS group was significantly lower than that in the Control group on POD-1 and POD-2 respectively [POD-1: 1.77(0.91) vs. 2.25(0.71); POD-2: 1.55(0.59) vs. 2.00(0.91); *P* < 0.05, respectively]. We also observed a lower VAS score trend in pTEAS-treated patients from POD-3 to POD-5, although this result did not show statistical significance (Table 3).

**Table 3:** Postoperative VAS pain score between two groups

	Con group (n = 40)	pTEAS group (n = 44)	P-value
T0	1.20(0.94)	1.09(0.86)	0.579
T1	2.25(0.71)	1.77(0.91)	0.009^a^
T2	2.00(0.91)	1.55(0.59)	0.007^a^
T3	1.80(1.14)	1.45(0.73)	0.098
T4	1.55(0.88)	1.55(1.04)	0.983
T5	1.45(0.93)	1.36(1.04)	0.69

Abbreviation: VAS = Visual analogue scales

a:P < 0.05 vs. control group.T0, baseline; T1,1 day after surgery; T2,2 day after surgery; T3,3 day after surgery; T4,4 day after surgery; T5,5 day after surgery

#### (iii) PONV and dizziness and consumption of antiemetics

**Fig. 3:**
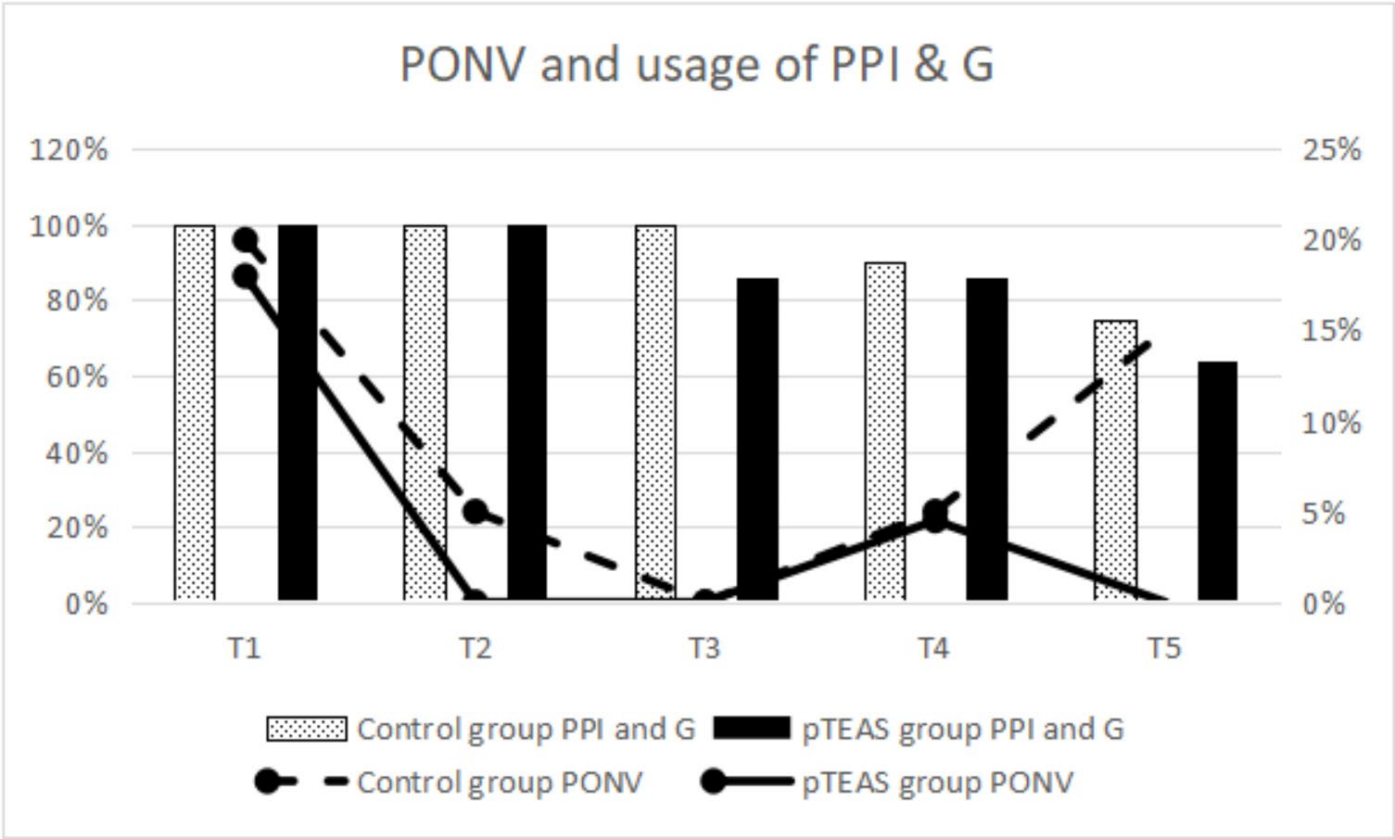
PONV and usage of PPI & G

Postoperative nausea and vomiting (PONV)—itself a distressing experience to patients—is a common side effect of surgery and anesthesia. Besides PONV, patients are also at risk of other discomforts such as aggravation of pain resulting from wound dehiscence. PONV is defined as nausea, retching, or vomiting, whereas dizziness is defined as disorientation in space, lightheadedness, or a sense of unsteadiness. At first, the rate of PONV from POD-1 to POD-4 and usage of rescue antiemetics from POD-1 to POD-5 were similar between groups. However, on POD-5, the rate of PONV was observed to be significantly lower in the pTEAS group than in the Control group [POD-5: 0 vs. 6(15%), P < 0.05]. The rate of dizziness on POD-1 and POD-5 was also significantly lower in pTEAS group [POD-1: 0 vs. 6(15%); POD-5: 2(4.5%) vs. 10(25%); P < 0.05, respectively] (Table 4), (Fig. 3).

**Table 4:** PONV and postoperative dizziness and usage of medication

	T1	T2	T3	T4	T5
PONV					
Control group (n = 40)	8(20%)	2(5%)	0	2(5%)	6(15%)
pTEAS group (n = 44)	8(18%)	0	0	0	0^a^
P values	0.832	0.224	/	0.224	0.009
Dizziness					
Control group (n = 40)	6(15%)	2(5%)	4(10%)	8(20%)	10(25%)
pTEAS group (n = 44)	0^a^	2(4.5%)	6(13.6%)	2(4.5%)^a^	2(4.5%)^a^
P values	0.009	1.000	0.741	0.042	0.011
PPI and G					
Control group (n = 40)	40(100%)	40(100%)	40(100%)	36(90%)	30(75%)
pTEAS group (n = 44)	40(100%)	40(100%)	38(86%)^a^	38(86%)	28(64%)
P values	/	/	0.027	0.741	0.261
Rescue Antiemetic					
Control group (n = 40)	4(10%)	2(5%)	0	0	0
pTEAS group (n = 44)	2(4.5%)	0	0	0	0
P values	0.418	0.224	/	/	/

Abbreviation: PONV, postoperative nausea and vomiting; PPI, Proton pump inhibitor; G, glucocorticoid

Data presented as n (%). a:P < 0.05 vs. control group

T1, 1 day after surgery; T2, 2 days after surgery; T3, 3 days after surgery; T4, 4 days after surgery; T5, 5 days after surgery

### Secondary outcome

#### (i) Perioperative hemodynamics

We analyzed the following data to understand perioperative hemodynamics and surgical stress: (1) blood pressure including SBP and DBP; (2) heart rate; and (3) NTI at the time of intubation, the start of the operation, end of the operation, and after tracheal extubation. Interestingly, the heart rate of patients in the pTEAS group was significantly lower than that of the Control group at the end of the operation [64.05(10.52) vs. 57.05(08.12) bpm; *P* < 0.05] and after tracheal extubation [78.70(15.28) vs. 69.09(11.00) bpm; *P* < 0.05] respectively. However, no significant difference was observed in intraoperative blood pressure and NTI between both groups (Table 5).

**Table 5:** Perioperative Systolic blood pressure (SBP), Diastolic blood pressure (DBP), heart rate (HR) and Narcotrend index (NTI).

	Control group (n = 40)				pTEAS group (n = 44)			
	SBP	DBP	HR	NTI	SBP	DBP	HR	NTI
T0	141.45(17.54)	83.70(10.80)	80.30(12.04)	98.40(00.59)	138.27(17.82)	83.00(09.09)	76.18(11.93)	98.36(00.78)
T1	111.75(26.36)	69.65(14.62)	75.30(16.71)	44.30(08.00)	105.95(15.50)	66.45(12.00)	74.86(13.84)	43.82(08.73)
T2	119.75(19.59)	70.25(18.58)	58.50(05.94)	40.45(06.35)	115.64(16.20)	72.00(10.15)	58.55(11.23)	39.64(04.97)
T3	129.10(13.36)	82.20(08.96)	64.05(10.52)	72.95(07.77)	132.68(15.33)	80.64(08.40)	57.05(08.12)^a^	75.82(10.02)
T4	129.90(16.10)	82.35(12.01)	78.70(15.28)	/	133.18(15.74)	80.32(09.49)	69.09(11.00)^a^	/

a:P < 0.05 vs. control group

T0, baseline; T1,at the time intubation; T2,at the start of surgery; T3,at the end of surgery; T4,at the time extubation

### (ii) Intraoperative blood loss

Perioperative bleeding poses a major surgical risk factor during both perioperative and postoperative periods; this could potentially result in complications, a high mortality rate, and a greater healthcare burden. We calculated the total volume of blood loss at the end of the operation and found that intraoperative blood loss was significantly lower in the pTEAS group in comparison to the Control group [379(202) *vs*. 497(283) ml, *P* < 0.05] (Table 2).

## Discussion

Our research showed that the cumulative dosage and index of remifentanil consumption in the pTEAS group were significantly lower than that in the Control group [Cumulative dosage: 1383(494) *vs*. 1637(630) µg; Index: 0.084(0.018) *vs*. 0.114(0.090) µg/min/kg; *P* < 0.05, respectively]. Remifentanil, a selective µ-receptor agonist commonly used in anesthesia, was found to be a risk factor for increased surgical site infection in colorectal surgery [23]. Additionally, remifentanil, along with other common opioids including morphine and fentanyl, has been shown to aggravate immune suppression after surgery [23–25]. In an RCT study conducted by Wang *et al.*, TEAS significantly reduced the dosage of remifentanil required during operation and the incidence of dizziness [20]. Our study demonstrated that lesser remifentanil was consumed due to the effect of pTEAS. This suggests that pTEAS can relieve pain, lower opioid consumption in surgeries, and alleviate anesthesia-related side effects.

Postoperative pain usually arises from the invasive and traumatic surgical process; in addition, it imposes a healthcare burden because its management requires extensive time and resources[26]. Statistics revealed that three in every four patients suffered from moderate to severe pain after an operation [27]. This type of pain demands medical attention because it can adversely affect the patient’s state of health by causing a slower recovery, a greater reliance on pain medications, and severe mental distress [28]. Hence, postoperative management of acute pain is crucial due to its effect on long-term clinical outcomes. We observed the patients until the postoperative day (POD) 5. According to our findings, the VAS score in pTEAS group was significantly lower than that in the Control group on POD-1 and POD-2, respectively [POD-1: 1.77(0.91) *vs*. 2.25(0.71); POD-2: 1.55(0.59) *vs*. 2.00(0.91); *P* < 0.05, respectively]. We also observed a lower VAS score trend in pTEAS-treated patients from POD-3 to POD-5, indicating that pTEAS could be used to improve the short-term management of postoperative pain.

PONV is a common side effect of surgery and anesthesia. The use of volatile agents induced moderate to severe nausea and vomiting in 30–50% of all general anesthetized patients [29]. A prospective survey revealed that surgical patients are often worried about PONV and its occurrence usually causes patient dissatisfaction [30]. PONV is an uncomfortable experience on its own, but it may even lead to wound dehiscence, water and electrolyte imbalance, or aspiration pneumonia [31]. A meta-analysis of 14 RCTs suggested that TEAS can effectively prevent PONV and dizziness [21]. Most studies on the incidence of PONV and dizziness were only from postoperative data recorded in 24 h [32]. However, the fact remains that PONV can last up to several days in high-risk patients. Our study demonstrated that PONV and dizziness continue to affect the patients at the end of the five-day postoperative follow-up; although patients in the pTEAS group experienced significantly less PONV (on POD-5) and dizziness (on POD-1 and POD-5) than patients in Control group. This shows that spinal surgery-related side effects such as PONV and dizziness can plague the patients for a long time after the operation. Therefore, it is critical to recognize ways of preventing and treating these side effects in a timely and effective manner. In terms of the patients covered by our study, the usual postoperative management of major spinal surgery entails giving a standard amount of glucocorticoid and proton pump inhibitor (PPI) medication after the surgery (POD 1–3). The purpose of both is to prevent PONV, with patients asking for rescue antiemetic after suffering from severe nausea and vomiting. Therefore, a higher incidence of PONV did not increase the use rate of rescue antiemetic in the Control group. Table 4; Fig. 3 illustrate that as the amount of drug (glucocorticoid and proton pump inhibitor (PPI) medication) decreased from POD3, the feeling of nausea and vomiting tended to increase. This suggests that the anti-nauseatic effect of both drugs are being cleared from the system. However, interestingly, the level of nausea and vomiting dropped to 0 in the pTEAS group at POD 5 (Fig. 3, p < 0.05), indicating that pTEAS did have a long-lasting effect of preventing PONV in our patients.

Studies have shown that heart rate affects survival and clinical outcome to a great extent. For example, a preclinical study involving rats suggested that a higher heart rate during vascular surgery is associated with a greater risk of adverse outcomes [33]. An increase in heart rate and blood pressure is caused by surgery-induced stress, in which endocrine, metabolic, and immunologic pathways become activated [34]. In this research, the heart rate at the end of surgery [64.05(10.52) vs. 57.05(08.12) bpm; *P* < 0.05] and at the time of extubation [78.70(15.28) vs. 69.09(11.00) bpm, *P* < 0.05] were significantly lower in pTEAS group in comparison to the Control group. This suggests that pTEAS may help alleviate cardiac adverse effects. A study by Fang et al. demonstrated that using TEAS apart from general anesthesia may protect against myocardial ischemia, facilitate recovery of cardiac function, and prevent apoptosis of cardiomyocytes [35]. Our study suggests that pTEAS may provide protection for the myocardium due to a lower heart rate and its inhibitory effect on surgery-induced stress.

Blood loss is an issue every surgeon and anesthetist pays a great deal of attention to during surgeries. Perioperative hemorrhage is a major surgical risk factor during both the perioperative and postoperative periods; this could potentially lead to complications, a high mortality rate, and a greater healthcare burden [36–39]. A study involving 39,309 surgical patients demonstrated that moderate to severe anemia due to intraoperative hemorrhage is associated with a higher rate of intensive care admission after surgery, longer hospital stay, and increased in-hospital death rate [40]. Previous studies revealed that patients who underwent spinal fusion surgery lost 0.5-3 L of blood during surgery and 30% of them eventually required transfusion [12, 13]. Furthermore, patients who require a blood transfusion during surgery have been shown to need longer in-hospital care and a higher morbidity rate after surgery [41]. We have found that intraoperative blood loss in the pTEAS group was significantly lesser compared to the Control group [379(202) vs. 497(283) ml, *P* < 0.05]. This data indicated that pTEAS not only has a potential protective effect on blood loss but also helps prevent complications and further injury. However, the in-depth mechanism of pTEAS warrants further research.

Nevertheless, we are aware of the limitations of our research. Firstly, this is a single-center clinical trial with a limited number of patients; a large, multicenter study is necessary to demonstrate the effectiveness of pTEAS from a broader perspective that includes other spinal surgery procedures. Secondary, the addition of a study group to receive electrical stimulation on a non-meridian and non-acupoint area would better demonstrate the effectiveness of stimulating specific acupoints.

## Conclusion

Effective pain management assumes great significance for improving patients’ quality of life (QoL), especially after major spinal surgeries. We observed that pTEAS could improve short-term pain management and clinical outcomes in these patients. Therefore, according to our recommendation, clinicians should include pTEAS as a complementary technique for effective short-term pain management, especially in patients undergoing major surgeries.

## Electronic supplementary material

Below is the link to the electronic supplementary material.

Supplementary Material 1

## Data Availability

The datasets used and analysed during the current study are available from the corresponding author on reasonable request.

## List of abbreviations

pTEAS: Perioperative transcutaneous electrical acupoint stimulation
TEAS: Transcutaneous electrical acupoint stimulation
PONV: Postoperative nausea and vomiting
PPI: Proton pump inhibitor
RCTs: Randomized controlled trials
POD: Postoperative day
TCI: Target-Controlled Infusion
NTI: NarcoTrend Index
PACU: Post-anesthesia Care Unit
SBP: Systolic blood pressure
DBP: Diastolic blood pressure
VAS: Visual analogue scale
QoL: Quality of life

## References

[1] Fritzell P, Hagg O, Wessberg P, Nordwall A, Swedish Lumbar Spine Study G. Volvo Award Winner in Clinical Studies: Lumbar fusion versus nonsurgical treatment for chronic low back pain: a multicenter randomized controlled trial from the Swedish Lumbar Spine Study Group. Spine 2001;26(23):2521–32; discussion 2532–2524. 10.1097/00007632-200112010-00002 11725230

[2] Ferrell BA (1991). Pain management in elderly people. J Am Geriatr Soc.

[3] DALYs GBD, Collaborators H, Murray CJ, Barber RM, Foreman KJ, Abbasoglu Ozgoren A, Abd-Allah F, Abera SF, Aboyans V, Abraham JP (2015). Global, regional, and national disability-adjusted life years (DALYs) for 306 diseases and injuries and healthy life expectancy (HALE) for 188 countries, 1990–2013: quantifying the epidemiological transition. Lancet.

[4] Deyo RA, Loeser JD, Bigos SJ (1990). Herniated lumbar intervertebral disk. Ann Intern Med.

[5] North RB, Kidd DH, Zahurak M, James CS, Long DM (1993). Spinal cord stimulation for chronic, intractable pain: experience over two decades. Neurosurgery.

[6] Lee JK, Amorosa L, Cho SK, Weidenbaum M, Kim Y (2010). Recurrent lumbar disk herniation. J Am Acad Orthop Surg.

[7] Willner D, Spennati V, Stohl S, Tosti G, Aloisio S, Bilotta F (2016). Spine Surgery and Blood Loss: Systematic Review of Clinical Evidence. Anesth Analg.

[8] Weir S, Samnaliev M, Kuo TC, Ni Choitir C, Tierney TS, Cumming D, Bruce J, Manca A, Taylor RS, Eldabe S (2017). The incidence and healthcare costs of persistent postoperative pain following lumbar spine surgery in the UK: a cohort study using the Clinical Practice Research Datalink (CPRD) and Hospital Episode Statistics (HES). BMJ open.

[9] Smith JS, Saulle D, Chen CJ, Lenke LG, Polly DW, Kasliwal MK, Broadstone PA, Glassman SD, Vaccaro AR, Ames CP (2012). Rates and causes of mortality associated with spine surgery based on 108,419 procedures: a review of the Scoliosis Research Society Morbidity and Mortality Database. Spine.

[10] Kamel H, Johnston SC, Kirkham JC, Turner CG, Kizer JR, Devereux RB, Iadecola C (2012). Association between major perioperative hemorrhage and stroke or Q-wave myocardial infarction. Circulation.

[11] Lee MJ, Konodi MA, Cizik AM, Bransford RJ, Bellabarba C, Chapman JR (2012). Risk factors for medical complication after spine surgery: a multivariate analysis of 1,591 patients. The spine journal: official journal of the North American Spine Society.

[12] Yoshihara H, Yoneoka D (2014). Predictors of allogeneic blood transfusion in spinal fusion in the United States, 2004–2009. Spine.

[13] Berenholtz SM, Pronovost PJ, Mullany D, Garrett E, Ness PM, Dorman T, Klag MJ (2002). Predictors of transfusion for spinal surgery in Maryland, 1997 to 2000. Transfusion.

[14] Murray DJ, Pennell BJ, Weinstein SL, Olson JD (1995). Packed red cells in acute blood loss: dilutional coagulopathy as a cause of surgical bleeding. Anesth Analg.

[15] Daniels AH, Riew KD, Yoo JU, Ching A, Birchard KR, Kranenburg AJ, Hart RA (2008). Adverse events associated with anterior cervical spine surgery. J Am Acad Orthop Surg.

[16] Grabowski G, Cornett CA, Kang JD (2012). Esophageal and vertebral artery injuries during complex cervical spine surgery–avoidance and management. Qld Gov Min J.

[17] Aziz M (2012). Airway management in neuroanesthesiology. Anesthesiol Clin.

[18] Palumbo MA, Aidlen JP, Daniels AH, Bianco A, Caiati JM (2013). Airway compromise due to laryngopharyngeal edema after anterior cervical spine surgery. J Clin Anesth.

[19] Swann MC, Hoes KS, Aoun SG, McDonagh DL (2016). Postoperative complications of spine surgery. Best Pract Res Clin Anaesthesiol.

[20] Wang H, Xie Y, Zhang Q, Xu N, Zhong H, Dong H, Liu L, Jiang T, Wang Q, Xiong L (2014). Transcutaneous electric acupoint stimulation reduces intra-operative remifentanil consumption and alleviates postoperative side-effects in patients undergoing sinusotomy: a prospective, randomized, placebo-controlled trial. Br J Anaesth.

[21] Chen J, Tu Q, Miao S, Zhou Z, Hu S (2020). Transcutaneous electrical acupoint stimulation for preventing postoperative nausea and vomiting after general anesthesia: A meta-analysis of randomized controlled trials. Int J Surg.

[22] Marsh B, White M, Morton N, Kenny GN (1991). Pharmacokinetic model driven infusion of propofol in children. Br J Anaesth.

[23] Inagi T, Suzuki M, Osumi M, Bito H (2015). Remifentanil-based anaesthesia increases the incidence of postoperative surgical site infection. J Hosp Infect.

[24] Sacerdote P, Bianchi M, Gaspani L, Manfredi B, Maucione A, Terno G, Ammatuna M, Panerai AE (2000). The effects of tramadol and morphine on immune responses and pain after surgery in cancer patients. Anesth Analg.

[25] Beilin B, Shavit Y, Hart J, Mordashov B, Cohn S, Notti I, Bessler H (1996). Effects of anesthesia based on large versus small doses of fentanyl on natural killer cell cytotoxicity in the perioperative period. Anesth Analg.

[26] Sun Y, Gan TJ, Dubose JW, Habib AS (2008). Acupuncture and related techniques for postoperative pain: a systematic review of randomized controlled trials. Br J Anaesth.

[27] Gan TJ, Habib AS, Miller TE, White W, Apfelbaum JL (2014). Incidence, patient satisfaction, and perceptions of post-surgical pain: results from a US national survey. Curr Med Res Opin.

[28] Joshi GP, Ogunnaike BO (2005). Consequences of inadequate postoperative pain relief and chronic persistent postoperative pain. Anesthesiol Clin North America.

[29] Schoenfeld AJ, Nwosu K, Jiang W, Yau AL, Chaudhary MA, Scully RE, Koehlmoos T, Kang JD, Haider AH (2017). Risk Factors for Prolonged Opioid Use Following Spine Surgery, and the Association with Surgical Intensity, Among Opioid-Naive Patients. J bone joint Surg Am volume.

[30] Gan TJ, Diemunsch P, Habib AS, Kovac A, Kranke P, Meyer TA, Watcha M, Chung F, Angus S, Apfel CC (2014). Consensus guidelines for the management of postoperative nausea and vomiting. Anesth Analg.

[31] Myles PS, Williams DL, Hendrata M, Anderson H, Weeks AM (2000). Patient satisfaction after anaesthesia and surgery: results of a prospective survey of 10,811 patients. Br J Anaesth.

[32] Jokinen J, Smith AF, Roewer N, Eberhart LH, Kranke P (2012). Management of postoperative nausea and vomiting: how to deal with refractory PONV. Anesthesiol Clin.

[33] Kooij FO, Vos N, Siebenga P, Klok T, Hollmann MW, Kal JE (2012). Automated reminders decrease postoperative nausea and vomiting incidence in a general surgical population. Br J Anaesth.

[34] De Oliveira GS, Castro-Alves LJ, Ahmad S, Kendall MC, McCarthy RJ (2013). Dexamethasone to prevent postoperative nausea and vomiting: an updated meta-analysis of randomized controlled trials. Anesth Analg.

[35] Yang Y, Yang X, Dong Y, Chen N, Xiao X, Liu H, Li Z, Chen Y (2016). Transcutaneous electrical acupoint stimulation alleviates adverse cardiac remodeling induced by overload training in rats. J Appl Physiol.

[36] El-Hussuna A, Qvist N, Zangenberg MS, Langkilde A, Siersma V, Hjort S, Gögenur I (2018). No effect of anti-TNF-α agents on the surgical stress response in patients with inflammatory bowel disease undergoing bowel resections: a prospective multi-center pilot study. BMC Surg.

[37] Fang JQ, Zhou CL, Shao XM, Guo XQ, Zhang LL, Jin L (2011). [Cardiac protective effects of transcutaneous electrical acupoint stimulation combined with general anesthesia for controlled hypotension]. Zhongguo zhen jiu = Chinese acupuncture & moxibustion.

[38] Wu WC, Smith TS, Henderson WG, Eaton CB, Poses RM, Uttley G, Mor V, Sharma SC, Vezeridis M, Khuri SF (2010). Operative blood loss, blood transfusion, and 30-day mortality in older patients after major noncardiac surgery. Ann Surg.

[39] Smilowitz NR, Oberweis BS, Nukala S, Rosenberg A, Zhao S, Xu J, Stuchin S, Iorio R, Errico T, Radford MJ (2016). Association Between Anemia, Bleeding, and Transfusion with Long-term Mortality Following Noncardiac Surgery. Am J Med.

[40] Christensen MC, Dziewior F, Kempel A, von Heymann C (2012). Increased Chest Tube Drainage Is Independently Associated With Adverse Outcome After Cardiac Surgery. J Cardiothorac Vasc Anesth.

[41] Stokes ME, Ye X, Shah M, Mercaldi K, Reynolds MW, Rupnow MF, Hammond J (2011). Impact of bleeding-related complications and/or blood product transfusions on hospital costs in inpatient surgical patients. BMC Health Serv Res.

[42] Baron DM, Hochrieser H, Posch M, Metnitz B, Rhodes A, Moreno RP, Pearse RM, Metnitz P, European Surgical Outcomes Study group for Trials Groups of European Society of Intensive Care M (2014). European Society of A: Preoperative anaemia is associated with poor clinical outcome in non-cardiac surgery patients. Br J Anaesth.

[43] Seicean A, Alan N, Seicean S, Neuhauser D, Weil RJ (2014). The effect of blood transfusion on short-term, perioperative outcomes in elective spine surgery. J Clin Neurosci.

