# Supplementary material for: Perioperative transcutaneous electrical acupoint stimulation (pTEAS) in pain management in major spinal surgery patients
Source: BMC Anesthesiol. 2022 Nov 8;22:342. doi: 10.1186/s12871-022-01875-3 (PMC9641754; doi:10.1186/s12871-022-01875-3)
Supplement: Supplementary file 1 — Supplementary Material 1 [file 12871_2022_1875_MOESM1_ESM.docx]

**Table S1.** General usage of NSAIDs and opioid.

|  | T1 | T2 | T3 | T4 | T5 |
| --- | --- | --- | --- | --- | --- |
| NSAIDs |  |  |  |  |  |
| Control group (n = 40) | 38 (95%) | 40 (100%) | 39 (97.5%) | 33 (82.5%) | 34 (85%) |
| pTEAS group (n = 44) | 43 (97.7%) | 44 (100%) | 42 (95.5%) | 41 (93%) | 37 (84%) |
| P values | 0.603 | / | 1.000 | 0.182 | 0.908 |
| opioid |  |  |  |  |  |
| Control group (n = 40) | 4 (10%) | 0 | 1 (2.5%) | 2 (5%) | 2 (5%) |
| pTEAS group (n = 44) | 2 (4.5%) | 2 (4.5%) | 2 (4.5%) | 2 (4.5%) | 1 (2.3%) |
| P values | 0.418 | 0.495 | 1.000 | 1.000 | 0.603 |

Abbreviation: NSAIDs, nonsteroidal anti-inflammatory drugs.

Data presented as n (%). a:P<0.05 vs control group.

T1, 1 day after surgery; T2, 2 days after surgery; T3, 3 days after surgery; T4, 4 days after surgery; T5, 5 days after surgery.
